# Retinal Microvascular Characteristics and Outcomes in Hypertensive Disorders of Pregnancy

**DOI:** 10.7759/cureus.67043

**Published:** 2024-08-16

**Authors:** Keerti Wali, Subhashchandra R Mudanur, Magna M Kuruvila, Vivea N Nagdev

**Affiliations:** 1 Ophthalmology, Shri B. M. Patil Medical College, Bijapur Lingayat District Education (BLDE), Vijayapura, IND; 2 Obstetrics and Gynaecology, Shri B. M. Patil Medical College, Bijapur Lingayat District Education (BLDE), Vijayapura, IND

**Keywords:** fundoscopy, perinatal mortality, hypertensive retinopathy, pre-eclampsia, pregnancy induced hypertension

## Abstract

Background

Hypertensive disorders of pregnancy (HDP) is a continuum of chronic hypertension, gestational hypertension, preeclampsia, and eclampsia in increasing severity, associated with a higher risk of complicated pregnancies and poor neonatal outcomes. This multisystem involvement can be assessed by fundoscopy, which serves as an indicator for generalized microvascular abnormalities. Our study aims to evaluate the correlation of hypertensive retinopathy with the severity of HDP and maternal and fetal outcomes.

Materials and methods

The study was conducted at a tertiary care hospital in Vijayapura from October 2021 to March 2022 among admitted cases of HDP. Detailed history, blood pressure (BP) measurement, obstetric examination, and fundoscopy were performed for all cases. Patients were followed up until the 10th postnatal day. The mode of delivery, birth weight, gestational age at birth, and any other neonatal outcomes were noted.

Results

We included 94 preeclampsia/eclampsia patients with a median age of 23 years, 51 (54.3%) being primigravida. Patients with chronic hypertension, gestational hypertension, and chronic hypertension superimposed by preeclampsia/eclampsia were excluded. The most common symptom in mothers was headache (23.4%), followed by blurring of vision (20.2%) and epigastric pain (5.3%) with a significant association (p < 0.05). Thirty-two cases (34%) had preterm deliveries with a positive association with the severity of retinopathy (p < 0.05). The magnitude of hypertensive retinopathy was 56.3% (53 cases), the severity of which significantly correlated to the severity of HDP (p < 0.05). We report 8.5% neonatal mortality and 22.3% small for gestational age (SGA) with a positive association with HDP severity (p < 0.05). There was no correlation between serum creatinine levels and the severity of retinopathy and fetal outcome.

Conclusion

The occurrence and severity of hypertensive retinopathy increase with increasing severity of HDP. Complaints, such as headache, blurred vision, and epigastric pain, are reported higher in cases with retinopathy. The severity of retinopathy may be used as an indicator of fetal morbidity; however, studies with large sample sizes and advanced tools are required to quantify the cause-effect relationship. The retinopathy associated with HDP resolves naturally with BP control postnatally.

## Introduction

Hypertensive disorders of pregnancy (HDP), also referred to as toxemia of pregnancy, is a multisystem disorder induced by pregnancy. Increased BP that occurs after 20 weeks of gestation in the absence of other causes of elevated BP (more than 140/90 mmHg measured twice with at least six-hour intervals) is defined as gestational hypertension [1]. Gestational hypertension, along with generalized edema and/or proteinuria (>300 mg per 24 hours), is defined as preeclampsia. Uncontrolled preeclampsia, eventually leading to seizures and/or coma, is eclampsia [1]. Thus, HDP is a continuum of gestational hypertension, preeclampsia, and eclampsia. Worldwide incidence of HDP is 4.6% [2], with prevalence ranging from 2% to 10%. The incidence of HDP in India is 5.4% [3].

A higher risk of complications, such as stillbirth, fetal growth restriction, bronchopulmonary dysplasia, acute respiratory distress syndrome, hematological complications like thrombocytopenia, neutropenia, intraventricular hemorrhage, sepsis, and neuro-psychomotor developmental disorders, are noted in neonates born to preeclamptic mothers [4]. Volhard, in 1918, proposed generalized vasospasm as the basic pathophysiology of HDP based on direct observation of small blood vessels in nail beds, ocular fundus, and bulbar conjunctiva [5]. Histopathological studies have further established this theory [6]. Vascular constriction leads to increased arterial hypertension by increasing blood flow resistance, thus inducing end-organ hypoxia, including placenta, liver, heart, kidneys, brain, and eyes. This multiorgan involvement can be directly assessed and monitored by visualizing the ocular fundus, which serves as an indicator of microvascular changes in other organs.

Numerous researchers have independently studied retinal microvascular characteristics, pregnancy complications, and neonatal complications of HDP. However, literature regarding the interplay between these factors is limited. The current study aims to assess the correlation of hypertensive retinopathy with the severity of HDP and maternal and fetal outcomes. This study is an attempt to evaluate whether microvascular characteristics of the retina serve as a predictor for fetal outcome in terms of gestational age, age-adjusted birth weight, and perinatal mortality.

## Materials and methods

Study design

This is a prospective observational study.

Place and duration of study

The study was conducted at Bijapur Lingayat District Education Deemed to be University (BLDEDU) Shri B. M. Patil Medical College and Research Centre, Vijayapura, Karnataka, from October 2021 to March 2022.

Sample size calculation

With a 40% anticipated proportion of fundus changes in the clinical diagnosis of HDP patients [7], the study requires a sample size of 93 patients at a 95% level of confidence and 10% absolute precision.

Inclusion and exclusion criteria

Inpatient pregnant women diagnosed with preeclampsia and eclampsia were included in the study. Gestational hypertension, by definition, does not involve target organs [1]. Hence, cases without proteinuria were excluded from this study to prevent a false low magnitude of hypertensive retinopathy. Cases with chronic hypertension and diabetes were excluded as they are independent risk factors for retinal changes. Also, cases with hazy ocular media and poor fundus visualization were excluded from the study. The institutional ethical committee approved our study.

Obstetric examination

After obtaining informed consent, an obstetrician conducted a detailed history and general physical and systemic examinations. Age, gravida, para, and BP were noted down. The diagnosis of preeclampsia was confirmed by raised BP (>140/90 mmHg) and proteinuria after 20 weeks of gestation. Along with routine hematological investigations, serum creatinine assessment was also done. Patients with pre-existing hypertension, diabetes mellitus, and renal disease are excluded from the study.

HDPs are classified as per ACOG guidelines as follows [8].

Preeclampsia: Systolic BP (SBP) of ≥140 mmHg or diastolic BP (DBP) ≥ 90 mmHg on two occasions at least four hours apart and proteinuria.

Preeclampsia with severe features: SBP ≥ 160 mm Hg or DBP ≥ 110 mm Hg and proteinuria along with thrombocytopenia, renal insufficiency, pulmonary edema, impaired liver function, or new-onset headache with or without visual disturbance.

Eclampsia: Preeclampsia with seizures.

Ophthalmic examination

Thorough ocular history was obtained, and anterior segment examination by hand-held slit lamp biomicroscope was performed by a single senior resident ophthalmologist to prevent observer bias. Tropicamide 1% eye drops were instilled three times every 10 minutes for pupillary dilatation. An ocular fundus examination was performed using indirect ophthalmoscopy. Hypertensive retinopathy changes seen in either or both eyes were taken as positive.

Hypertensive retinopathy was graded according to the Keith-Wagener classification [9] into the following.

Grade 1: Mild generalized arterial attenuation.

Grade 2: More severe grade 1 and focal arteriolar attenuation.

Grade 3: Grade 2 + hemorrhages, hard exudates, cotton wool spots.

Grade 4: Grade 3 + optic disc swelling (papilledema).

The eye with a higher grade was considered in case of asymmetrical involvement. Ophthalmoscopy was performed every day till the 10th postnatal day. The mode of delivery, whether cesarean, spontaneous vaginal, or induced vaginal delivery, was noted. Fetal outcomes were evaluated in terms of appropriate for gestational age (AGA), small for gestational age (SGA), and perinatal death.

Statistical analysis

The data obtained were entered in a Microsoft Excel sheet (Microsoft® Corp., Redmond, WA), and statistical analysis was performed using Statistical Package for the Social Sciences (IBM SPSS Statistics for Windows, IBM Corp., Version 26, Armonk, NY). Results were presented as mean ± SD, median and interquartile range, frequency, percentages, and diagrams. The significant difference in categorical variables was compared using the chi-square test. Quantitative data were analyzed using an independent t-test and ANOVA test; p < 0.05 was considered statistically significant. All statistical tests were performed in two-tailed.

## Results

**Figure 1:**
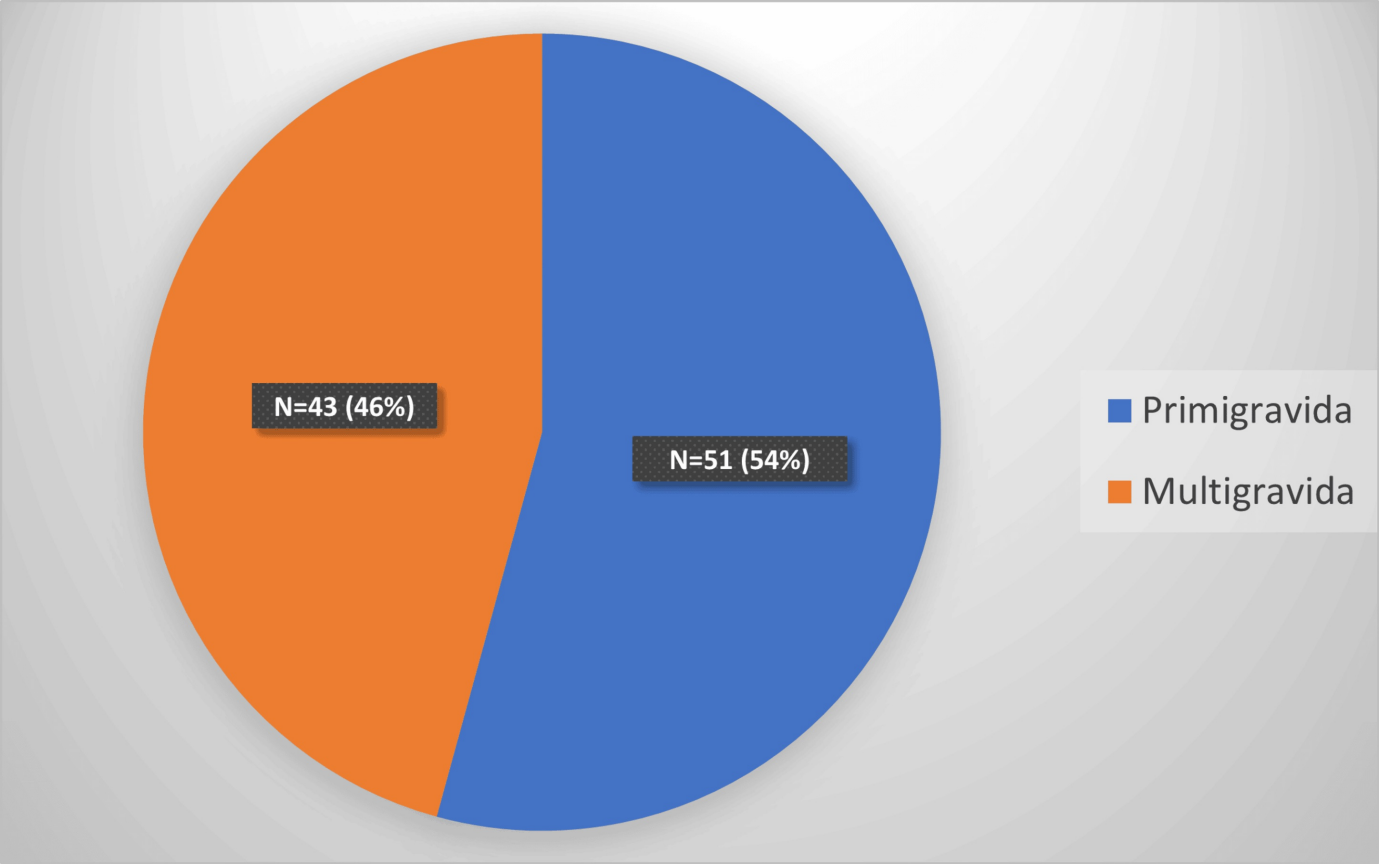
Gravida status of cases

**Figure 2:**
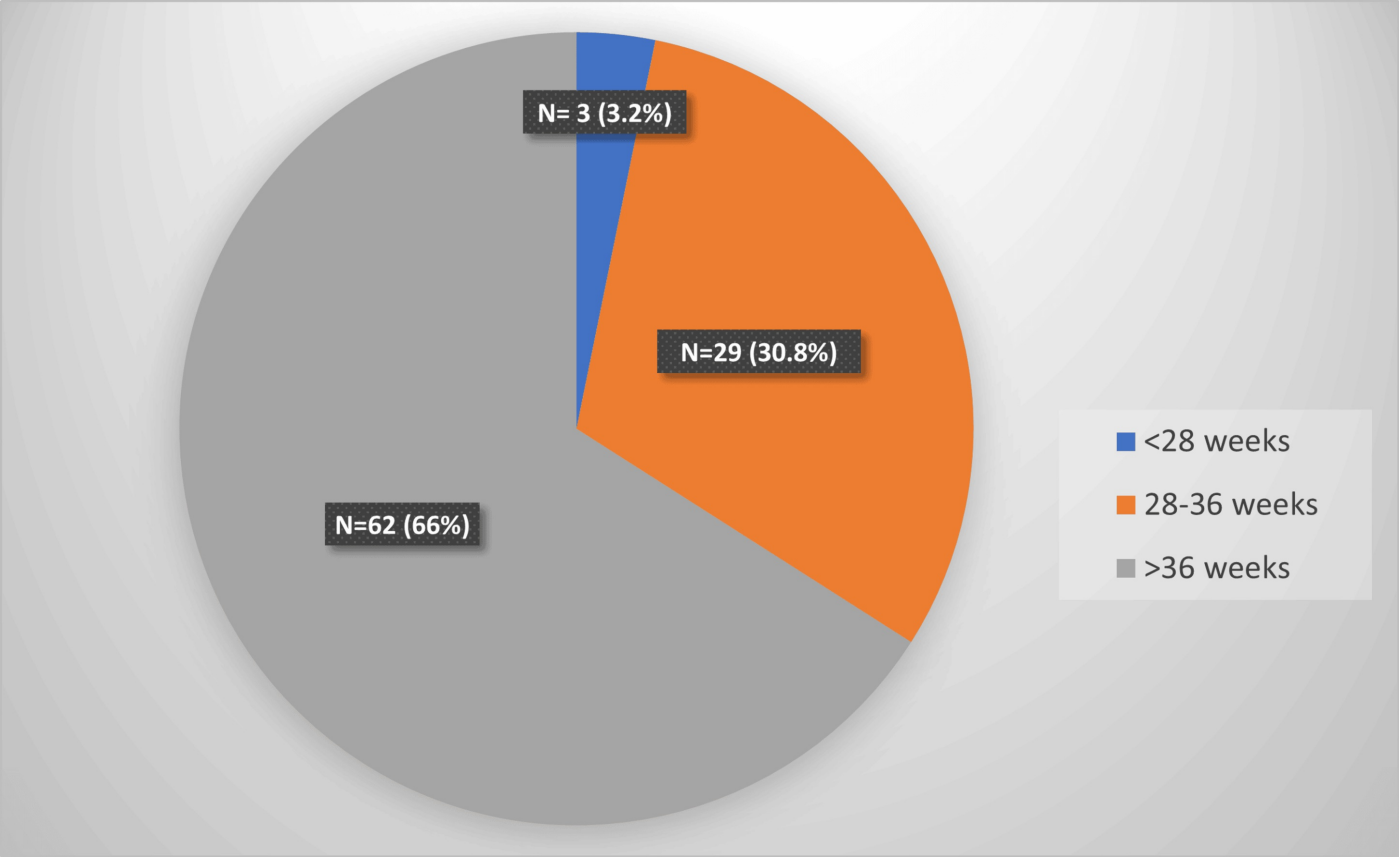
Gestational age at the time of delivery

The current study included 94 HDP patients ranging from 18 to 35 years of age with a median age of 23 years. We included 51 primigravida (54.3%) and 43 (45.7%) multigravidas (Figure 1). The most common symptom in mothers was headache (23.4%), followed by blurring of vision (20.2%) and epigastric pain (5.3%) (Table 1). The association of the occurrence of these symptoms with the severity of HDP was statistically significant by the chi-square test (p = 0.035). Sixty-two cases (66%) delivered at term, 29 cases (30.8%) between 28 and 36 weeks, and three cases (3.2%) delivered before 28 weeks (Figure 2). There was no statistically significant association (chi-square test, p = 0.19) between the presence of hypertensive retinopathy and preterm deliveries (Table 2).

**Table 1:** Symptoms associated with pregnancy-induced hypertension PE, preeclampsia Chi-square test, p = 0.035

	Headache	Blurring of vision	Epigastric pain	Nil	Total
Mild PE	6	4	2	15	27
Severe PE	8	9	1	30	48
Eclampsia	8	6	2	3	19
Total	22	19	5	48	94

**Table 2:** Association of preterm delivery with hypertensive retinopathy Chi-square test, p = 0.19

	Grade of hypertensive retinopathy					Total
	0	1	2	3	4	
Preterm deliveries	11	10	7	2	2	32
Term deliveries	30	23	7	2	0	62
Total	41	33	14	4	2	94

**Figure 3:**
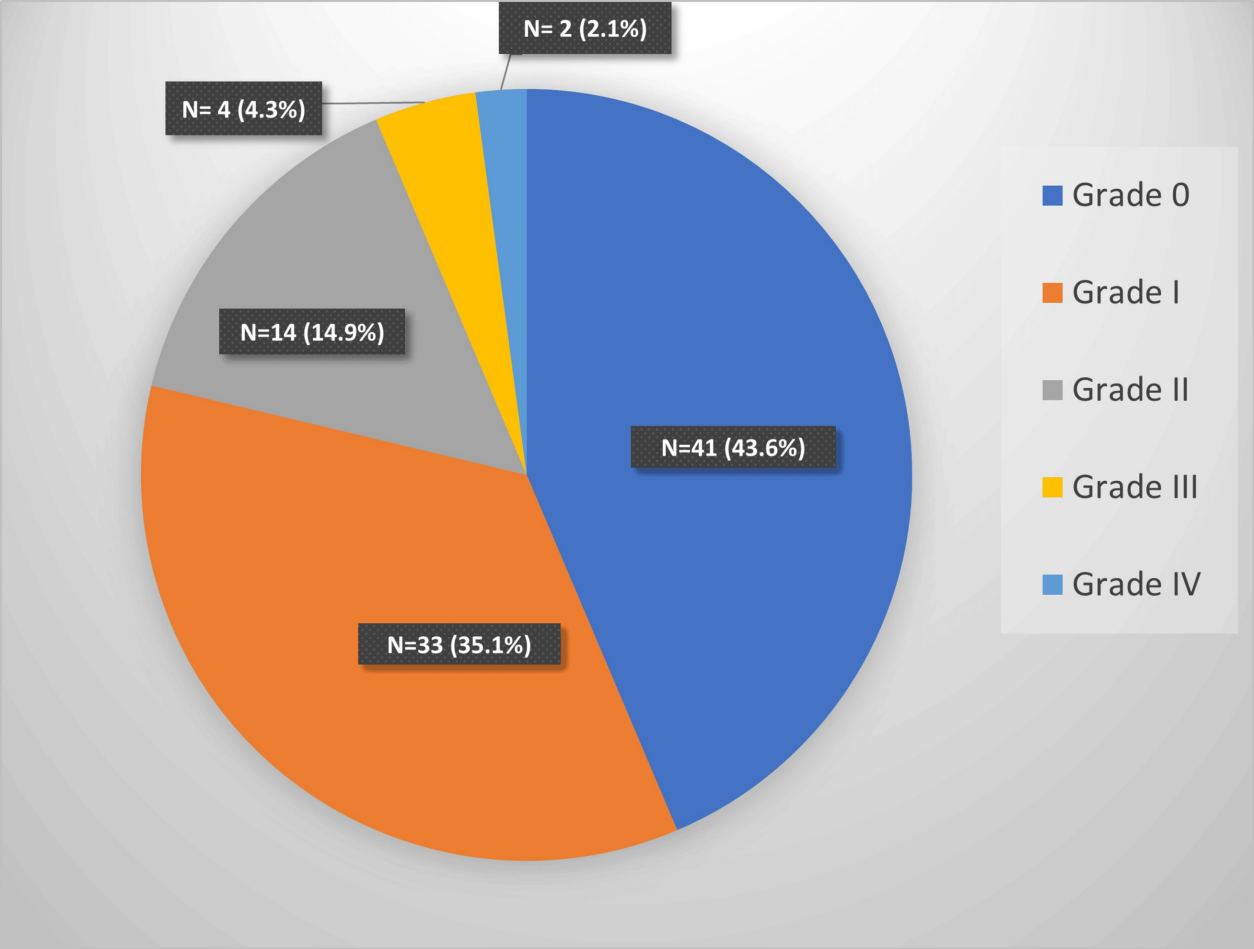
Hypertensive retinopathy changes in cases as per Keith-Wegener's classification

Fifty-three (56.3%) cases were diagnosed as having hypertensive retinopathy, with 33 (35.1%) patients having grade 1 retinopathy (Figure 3). Twenty-seven cases (28.7%) cases were diagnosed as preeclampsia, 48 cases (51.1%) as preeclampsia with severe features, and 19 cases (20.2%) as eclampsia. The severity of hypertensive retinopathy showed a significant correlation by chi-square test with the severity of HDP (Table 3).

**Table 3:** Severity of hypertensive retinopathy versus severity of hypertensive disorders of pregnancy Chi-square test, p = 0.0018

	Hypertensive retinopathy grades					Total
	0	1	2	3	4	
Preeclampsia	20	7	0	0	0	27
Preeclampsia with severe features	15	19	11	3	0	48
Eclampsia	6	7	3	1	2	19
Total	41	33	14	4	2	94

The decision to induction of lower segment cesarean section was taken for various obstetrics indications. Seventy-three patients (77.7%) underwent lower-segment cesarean section, and 21 (22.3%) underwent vaginal delivery.

While 65 mothers were blessed with AGA neonates, 21 neonates were SGA, and eight mothers unfortunately either had an intrauterine death (IUD) or neonatal death. The association of fetal outcome with retinal microvascular changes was significant by chi-square test (p = 0.003) (Table 4).

**Table 4:** Association of retinal changes with fetal outcome AGA, appropriate for gestational age; SGA = small for gestational age Chi-square test, p = 0.0017

Grade of hypertensive retinopathy	AGA	SGA	Perinatal death	Total
0	37	4	0	41
1	17	12	4	33
2	10	2	2	14
3	1	2	1	4
4	0	1	1	2
Total	65	21	08	94

Serum creatinine levels were similar in all grades of HDP. The average level was 1.16 ± 0.46 mg/dL in total sample size, which did not differ much in patients with and without fundus changes. There was no statistically significant correlation between creatinine levels and fetal outcome (Table 5).

**Table 5:** Serum creatinine values in hypertensive disorders of pregnancy Reference values for serum creatinine = 0.7-1.3 mg/dL AGA, appropriate for gestational age; SGA, small for gestational age

Sub-group	Serum creatinine levels, mean ± SD in mg/dL	Number of cases (n)	Significance
Total sample	1.16 ± 0.46	94	-
Cases without hypertensive retinopathy	1.14 ± 0.45	41	Independent t-test, p = 0.79
Cases with hypertensive retinopathy	1.16 ± 0.48	53	
Cases with AGA neonate	1.15 ± 0.47	65	ANOVA test, p = 0.98
Cases with SGA neonate	1.16 ± 0.47	21	
Cases with perinatal death	1.16 ± 0.47	08	

Post-delivery, BP decreased in 89 cases (94.7%), while five cases showed persistently elevated BP even at 10 days follow-up. BP control was coupled with the improvement of hypertensive retinopathy in 44 (83%) cases out of 53 patients, whereas the remaining nine cases did not show improvement by the 10th postnatal day.

## Discussion

Preeclampsia is a pregnancy-specific multisystem disorder characterized by hypertension after 20 weeks gestation. In this enigmatic conundrum of HDP, it is a demystified fact that the disease contributes to gross maternal vascular endothelial dysfunction, vasospasm, and capillary leak. The altered vascular dynamics lead to cardiovascular changes, hematological changes, impairment in renal and hepatic systems, and cerebral and neurological manifestations [10]. Vessels of women with preeclampsia show hypersensitivity to vasopressors and decreased response to vasodilators and vascular levels of vasodilators such as nitric oxide and prostacyclin [11]. Clinical evidence of these microvascular characteristics can be obtained by fundus examination.

Interpretation

The current study included 94 HDP patients with a median age of 23 years. Most of the cases (51.1%) had a severe grade of preeclampsia. This could be a sampling bias as it was a hospital-based study, and mild cases are usually managed on an outpatient basis. Our study included 54.3% primigravida cases, which is comparable to 60% in a study by Ranjan et al. [7].

Our study demonstrated 34% preterm deliveries and a higher rate of retinopathy (65.6%) compared to term deliveries with 51.6% retinopathy, although statistically insignificant. Our results are congruent with earlier findings by Bakhda et al. [12], who reported 72.5% positivity. Thus, our study, along with many others, suggests a significant increase in the rate of preterm delivery as the severity of retinopathy increases [12-14]. The presence of retinopathy indicates a multisystem vascular compromise and influences obstetricians to decide on early termination of pregnancy, thus increasing rates of preterm deliveries. Grade 4 hypertensive retinopathy changes like serous retinal detachment and papilledema are absolute indications of pregnancy termination. Since cesarean section is the most commonly preferred mode of delivery in complicated circumstances in fear of fetal distress and the presence of comorbidities, the rate of delivery by cesarean section is higher in our study (77.7%).

Ocular involvement is very common in HDP [15]. Blurring of vision, either sudden or gradual, photopsia, scotoma, and diplopia are the most common symptoms. Visual symptoms are considered to be precursors of seizures [15]; 20.2% of our cases complained of blurring of vision compared to 12.2% in a study by Ranjan et al. [7]. Bakhda et al. reported all patients with visual complaints to have positive retinopathy features, possibly owing to macular edema, whereas only 14 of our 19 patients with visual complaints were positive for retinopathy. We believe milder grades of retinopathy may not lead to visual symptoms. Also, the authors may have used advanced techniques for fundus examination, like optical coherence tomography, unlike our study, where retinopathy was diagnosed only by indirect ophthalmoscopy.

Headache was the most commonly reported complaint by our study recruits (24.4%). Cases also complained of epigastric pain, a symptom often associated with severe preeclampsia; 51% of patients were asymptomatic, similar to observations by Ranjan et al. [7], who reported 36% cases with headache and 52% asymptomatic patients.

Fundus examination revealed retinopathy changes in 56.4% of cases, 62.3% of them having grade 1 hypertensive retinopathy, grade 2 in 26.4%, grade 3 changes in 7.6%, and only 3.8% with grade 4 retinopathy. The results are consistent with the studies by Kamath et al. [13] and Bakhda et al. [12], with 60% and 51% incidence of retinopathy changes, respectively. The literature review indicates a wide range of incidences of retinopathy, from 13.7% [16] to 60% [7,13,17]. Severe arteriolar spasm is the most common finding present in 70% of cases of preeclampsia [18]. A hundred percent incidence of hypertensive retinopathy has been reported at systolic BP > 180 mmHg and diastolic BP > 120 mmHg [12]. The incidence of retinopathy is said to increase with the severity of HDP [12,13,19], statistically illustrated in our study. Jacob et al. [19] suggested using retinopathy as a marker for the severity of HDP. The degree of dilatation and constriction of the retinal microvasculature, even during normal pregnancy, have been shown to correlate with the physiological changes in the mean arterial BP [20].

The severity of retinopathy was negatively associated with fetal outcome. Varying degrees of retinopathy were observed in 100% of cases of perinatal death and 81% of SGA cases. Kamath et al. [13] reported a very high incidence of 100% and 81.8% of SGA and perinatal mortality, respectively, in cases with grades 2 and 3 retinopathy. Bakhda et al. [12] reported 23% stillbirth cases with 60% positivity for retinopathy changes. Impaired extravillous trophoblast invasion of the spiral arteries in HDP results in failure of vascular remodeling and dilatation, leading to placental under-perfusion [21]. Ischemic theory has been postulated to cause low birth weight in pregnancy-induced hypertension (PIH) [7,14,19]. However, this concept has been questioned by other authors [22,23], who propose birth weight as a function of gestational age. They believe uterine blood flow in HDP is increased secondary to increased cardiac output. A study by Obed et al. [24] noticed lower birth weight in early-onset HDP compared to late-onset HDP, as the former is severe and long-standing.

Serum creatinine levels were tested in all patients, and no difference was noted in our study between different grades of retinopathy and different fetal outcomes. Previous studies have demonstrated increasing creatinine values with increased severity of retinopathy [19,25]. The authors hypothesize that the elevated levels may be due to decreased urinary clearance secondary to reduced glomerular filtration rate and increased reabsorption. Our results are different, possibly owing to an evolving healthcare system, which aids timely intervention to control BP with reduced possibility of renal dysfunction.

BP reduction was noted in 94.7% of patients postnatally, with 83% of cases of retinopathy showing improvement by day 10. Kamath et al. [13] reported 95% improvement in retinopathy. Vasospastic manifestations are reversible, and retinal vessels rapidly return to normal after delivery [15]. Patients and their caregivers must be reassured about visual symptoms. BP control, safe delivery, and exceptional neonatal care should be a prime focus during the management of these cases.

Clinical implications

Fundus examination not only helps in diagnosis but also in the progression of disease, assessment of treatment response, and prediction of the ultimate prognosis. Hence, the ophthalmoscope, second to the sphygmomanometer, is the most important instrument in HDP case evaluation, and the services of an ophthalmologist are essential in the proper conduct of the obstetrics department.

Limitations of study

The retinal microvascular examination technique used in our study is very primitive in an era of advanced ophthalmic imaging techniques. The generalization of results is questionable, owing to the smaller sample size. Further studies in a larger sample incorporating detailed retinal microvascular evaluation using optical coherence tomography angiography (OCTA) with quantified data are suggested to augment our knowledge.

## Conclusions

The occurrence and severity of hypertensive retinopathy increase with increasing severity of HDP. All cases complaining of blurring of vision may not clinically have hypertensive retinopathy. Complaints, such as headache, blurring of vision, and epigastric pain, are reported higher in cases with retinopathy. The severity of retinopathy may be used as an indicator of fetal morbidity. However, studies with large sample sizes and advanced tools are required to quantify the cause-effect relationship. The retinopathy associated with PIH resolves naturally with BP control postnatally.
